# Serum Copper Concentrations, Effect Modifiers and Blood Pressure: Insights from NHANES 2011–2014

**DOI:** 10.3390/jcdd10100432

**Published:** 2023-10-18

**Authors:** Ruo-Nan Xu, Yue Zhang, Xin Xu, Xu Li, Lan He, Qiang Feng, Yong-Hai Yang, Yang He, Xiao Ma, Yong-Ming He

**Affiliations:** 1 Division of Cardiology, The First Affiliated Hospital of Soochow University, 188 Shizi Street, Suzhou 215006, China; xuruonan19982021@163.com (R.-N.X.); 15905170271@163.com (Y.Z.); hsu18752789840@163.com (X.X.); xu_li1996@163.com (X.L.); helan0325@163.com (L.H.); fengqiangting@163.com (Q.F.); heyang0531@suda.edu.cn (Y.H.); 2 Division of Ultrasound, The Shanghai Municipal No 8 People’s Hospital, 8 Caobao Street, Shanghai 200000, China; 3 Division of Cardiology, Suzhou Yongding Hospital, 1388 Gaoxin Street, Suzhou 215299, China; 274096724@163.com

**Keywords:** copper, hypertension, blood pressure, antihypertensive drugs

## Abstract

(1) Background: Epidemiological studies on the relationship between serum copper and hypertension are contradictory. We assessed the relationship between serum copper and blood pressure among adults in the United States. (2) Methods: We divided hypertension into two categories: treated hypertension and untreated hypertension. Linear or logistic regression analysis was applied to investigate the association between serum copper concentrations and blood pressure levels. (3) Results: As compared to quartile 1, the odds ratios (ORs) for untreated hypertension in quartiles 2, 3, and 4 were 1.02 (0.74–1.42), 1.23 (0.88–1.72), and 1.08 (0.74–1.58), respectively, in multivariable analysis (all *p* > 0.05). In non-hypertension, as compared with quartile 1, the β (95% CI) of systolic blood pressure for quartiles 2, 3, and 4 was −0.92 (−2.07–0.23), −0.05 (−1.30–1.20), and −0.48 (−1.83–0.88), respectively, in multivariable analysis (all *p* > 0.05). As compared to quartile 1, the ORs for treated hypertension in quartiles 2, 3, and 4 were 1.36 (0.88–2.10), 1.35 (0.87–2.09), and 1.56 (0.98–2.47), respectively, upon multivariable analysis including antihypertensive medication use as a covariate (all *p* > 0.05). Furthermore, 1SD increase in serum copper was non-significantly associated with 1.16 (0.97–1.37)-fold increased risk of hypertension in multivariable analysis (*p* = 0.096). (4) Conclusion: In the present study, we discovered that the serum copper concentration was not related with hypertension or blood pressure levels. Antihypertensive drug use may distort the correlation between copper and blood pressure levels. Information on antihypertensive drug use may be taken into account when identifying new risk factors for hypertension.

## 1. Introduction

Hypertension, a global public health issue, is the principal reversible risk factor for cardiovascular disease (CVD) and the leading cause of mortality worldwide [1,2]. The control of hypertension is attributable to the prevention and treatment of conventional risk factors, such as, obesity, physical inactivity, and smoking [3]. Thus, it is crucial to examine less studied risk factors to control hypertension.

Recently, trace elements and their associations with hypertension have come into our view, although these associations are conflicting. Two prior studies reported significant associations of serum copper with hypertension, in which one study was in fact a univariate analysis without considering confounding factors [4]. In the other study, information on the antihypertensive drug use was missing, although the copper–hypertension association was examined using multivariable analyses [5]. Two more studies all from the NHANES data did not identify a serum copper–hypertension association in the multivariable analysis. Unfortunately, an important covariate of antihypertensive drug use was missing in one study [6]. Hypertension diagnosis in another study was based on SBP ≥ 130 mmHg and or DBP ≥ 80 mmHg, different from the acknowledged SBP ≥ 140 mmHg and or DBP ≥ 90 mmHg, which distorted the true associations of serum copper with high blood pressure [7]. Therefore, the above-mentioned studies’ inconsistent conclusions are due to their fallacies in study design, analysis methods, and varying criteria for hypertension diagnosis. In this study, we examined the association between serum copper and blood pressure among adults in the United States.

## 2. Materials and Methods

### 2.1. Study Population

**Figure 1:**
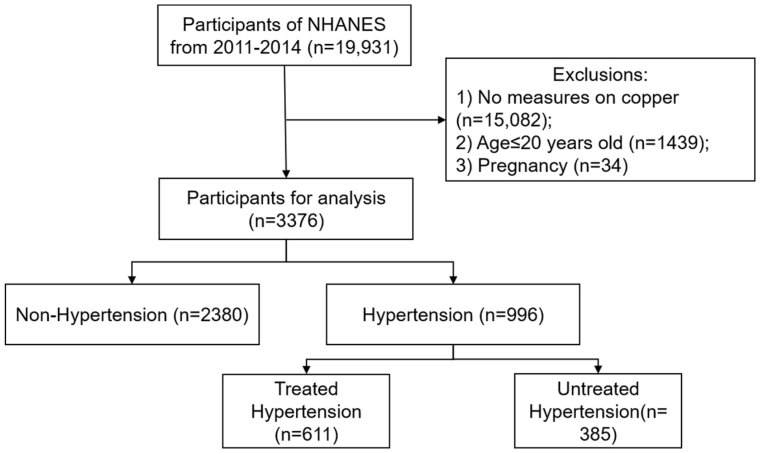
Flow chart of the patient selection.

Our analyses were based on the National Health and Nutrition Examination Survey (NHANES), which was a research project designed to assess the health and nutritional status of the US population [8]. For this study, two publicly accessible datasets from two survey cycles (2011–2012 and 2013–2014) were included. Participants with missing data on serum copper (*n* = 15082) were excluded. Adolescents younger than or 20 years of age (*n* = 1439) and pregnant women (*n* = 34) were also removed. Thus, 3376 eligible subjects were included for final analysis. The study population included non-hypertension (*n* = 2380) and hypertension (*n* = 996). We further classified hypertensive participants into treated hypertension (*n* = 611) and untreated hypertension (*n* = 385). The flowchart of patient selection is shown in Figure 1. All participants signed informed consent for NHANES.

### 2.2. Measurement of Serum Copper

All participants’ blood was collected in corresponding tubes and was gently inverted five to six times as soon as possible. After clotting for 30–45 min and centrifugation at 2900 rpm for 15 min both at room temperature, serum was stored in frozen conditions and transported to the Division of Laboratory Sciences, National Center for Environmental Health, Centers for Disease Control and Prevention, for analysis. The serum copper concentration was quantified using inductively coupled plasma dynamic reaction cell mass spectrometry (ICP-DRC-MS), a powerful multielement analytical technique with the ability to detect even trace levels of elements. All the data exceeded the lower limit of detection (LLOD) for the tests. The LLOD of serum copper was 2.5 μg/dL. More comprehensive information on laboratory procedures and methods is accessible through the official website: https://www.cdc.gov/nchs/nhanes/index.htm (accessed on 5 July 2023).

### 2.3. Measurement of Blood Pressure Levels and Diagnosis of Hypertension

Blood pressure was measured by trained and qualified staff according to standardized protocol and procedure at the mobile examination center. In simple terms, after the subjects were asked to sit quietly for 5 min, certified examiners measured their blood pressure and obtained three or sometimes four consecutive blood pressure readings. The average of all acquired measurements was calculated and recorded. Participants were defined as having hypertension if they satisfied one or more of the following conditions: (1) mean systolic blood pressure ≥140 mmHg, (2) mean diastolic blood pressure ≥ 90 mmHg, (3) subjects who were diagnosed to have high blood pressure, and (4) individuals with currently taking prescribed medicine for hypertension. Hypertension was further classified into 2 categories: hypertension under or not under treatment.

### 2.4. Antihypertensive Medications

Antihypertensive medications were determined according to the drugs reported by the patients to have been taken during the previous 30 days. According to the 2017 ACC/AHA hypertension guidelines, we categorized antihypertensive drugs as angiotensin-converting enzyme inhibitors (ACE-Is), angiotensin receptor blockers (ARBs), beta-blockers (BBs), calcium channel blockers (CCBs), thiazide and thiazide-like diuretics (thiazides), as well as others [9].

### 2.5. Covariates

Potential covariates associated with hypertension were determined according to previously published studies [7,10]. We evaluated whether there is a mediating effect of covariates using Baron and Kenny’s method [11]. These covariates included demographic information on age, sex, education levels, lifestyle information on smoking and drinking status, examination information on body mass index (BMI), laboratory indicators about the estimated glomerular filtration rate (eGFR), high-density lipoprotein cholesterol (HDL-C), as well as self-reported diseases like type 2 mellitus diabetes and coronary artery diseases (CAD). The education level was divided into three groups: below high school, high school, and above high school. Smoking status and drinking status were both categorized as never, former, and current. ‘Never smoking’ referred to someone who has not smoked over 100 cigarettes during their lifetime and does not smoke at present. ‘Former smoking’ was someone who has smoked over 100 cigarettes during their lifetime, but has not smoked within the past 28 days. ‘Current smoking’ was defined as a subject who has smoked over 100 cigarettes during their lifetime and has smoked at least once within the past 28 days. Never, former, and current drinking corresponded to the following situations, respectively, participants who drunk less than 12 drinks in their lifetime, individuals who drunk more than 12 drinks during their lifetime, but no drinks in the past year, and subjects who drunk in the past year. BMI was computed utilizing the formula weight (kg) divided by the square of height (m^2^). eGFR was computed employing the Chronic Kidney Disease Epidemiology (CKD-EPI) formula [12]. Diabetes was defined as glycated hemoglobin A1c ≥ 6.5% or fasting glucose ≥ 7 mmol/L or 2 h plasma glucose ≥ 11.1 mmol/L during an OGTT or the use of hypoglycemic medication [13].

### 2.6. Statistical Analysis

For descriptive analysis, continuous variables are shown as the mean ± standard deviation, and categorical variables are shown as a percentage (numbers). In particular, age is presented as the median (interquartile range, IQR) for its non-normal distribution. Differences in variables between quartiles of serum copper were analyzed using chi-square (categorical variables), ANOVA (continuous variables), and Kruskal–Wallis tests only for age. Alongside that, standardized serum copper was adopted in order to unify data and facilitate analysis.

First, logistic regression models were utilized to compute the odds ratios (ORs) and their respective 95% confidence intervals (CIs) to furnish a quantitative assessment of the association between serum copper and hypertension prevalence. We regarded subjects with untreated hypertension as cases, and those without hypertension were the controls. Then, linear regression models were employed to investigate the correlation between serum copper and systolic BP and diastolic BP in a non-hypertensive population. Testing for trends in event rates across the quartiles was performed using the STATA procedures Opartchi. We fitted 2 statistical models: the crude model was not adjusted and the adjusted model was adjusted for education, smoking status, drinking status, BMI, eGFR, HDL-C, diabetes status, and CAD. We performed multiple imputation for missing values of systolic and diastolic blood pressure, as well as all covariates under the assumption of missing at random, with 25 imputations [14].

Moreover, we carried out several tentative analyses. To assess the effect of different antihypertensive drugs on the relationship between serum copper and hypertension, a three-stage analysis was performed. First, we took participants with treated hypertension as cases and those with non-hypertension as controls. Second, the setting of cases and controls in the first stage was unchanged, and the multivariate model was further adjusted for antihypertensive medications. Third, we set hypertensive patients treated with single antihypertensive drugs as the case group, and the non-hypertensive subjects as the control group. Stratified analyses were implemented by sex (male, female), age (≤48, >48 y), BMI (<30, ≥30), smoking status (smoker, non-smoker), drinking status (drinker, non-drinker), education level (≤high school, >high school), eGFR (<60, ≥60 mL/min/1.73 m^2^), diabetes (Yes, No). The product terms between serum copper and stratification variables were introduced into the same regression model as covariables to perform interaction analysis. Moreover, we conducted a few sensitivity analyses to evaluate the soundness of the results. On the one hand, the models were reconstructed with excluding participants with impaired eGFR (<60 mL/min/1.73 m^2^) to account for the effect of renal function injury on blood pressure. On the other, we refitted our models after keeping serum samples in the range of the 1st to 99th percentiles of the serum copper distribution to eliminate the influence of outliers. Data analysis was conducted using STATA version 17.0 and R software version 4.2.0. All tests were two-sided, and a *p*-value < 0.05 was deemed to be significant.

## 3. Results

### 3.1. Baseline Characteristics

The characteristics of the study individuals according to quartiles of serum copper are depicted in Table 1. In two survey cycles, 3376 adults were included for final analysis, with 49.61% men. Among the participants, the median (IQR) age was 48 (34–63) years and the median (IQR) serum copper levels were 17.96 (15.59–21.02) μmol/L. The distribution of serum copper across the study is shown in Figure S1 in the Supplementary Materials. The subjects in the highest quartile of serum copper level tended to be older, female, and never-drinkers, as well as more inclined to possess a higher BMI, HDL-C, SBP, and lower DBP level. Additionally, we discovered a higher prevalence of diabetes in the subjects with higher serum copper level. Interestingly, participants with a higher serum copper level were inclined to have a higher prevalence of treated hypertension (*P* for trend < 0.001). In contrast, the prevalence of untreated hypertension varied weakly across serum copper quartiles.

### 3.2. Association of Serum Copper with Untreated Hypertension

The association between serum copper and untreated hypertension prevalence is depicted in Table 2. In multivariable analysis, as compared to quartile 1, the ORs for untreated hypertension in quartiles 2, 3, and 4 were 1.02 (0.74–1.42), 1.23 (0.88–1.72), and 1.08 (0.74–1.58), respectively. The P for trend was non-significant. The association of serum copper with untreated hypertension was also not revealed on a continuous scale.

### 3.3. Association of Serum Copper with Blood Levels for Non-Hypertension

In multivariable regression analysis, as compared with quartile 1, β (95% CI) of systolic blood pressure for quartiles 2, 3, and 4 was −0.92 (−2.07–0.23), −0.05 (−1.30–1.20), and −0.48 (−1.83–0.88), respectively (Table 3). On a continuous scale, the serum copper–blood pressure association was also not revealed. Similar results were demonstrated with respect to diastolic blood pressure.

### 3.4. Association of Serum Copper with Treated Hypertension

In crude analysis, on a categorical or continuous scale, serum copper levels were notably associated with hypertension in individuals receiving antihypertensives. In partially adjusted analysis without including antihypertensives as a covariate, this association remained. However, this association disappeared after further adjustment for antihypertensive drugs. As compared to quartile 1, the ORs for hypertension in quartiles 2, 3, and 4 were 1.36 (0.88–2.10), 1.35 (0.87–2.09), and 1.56 (0.98–2.47), respectively (Table 4). The P for trend was non-significant. Specifically, the association of serum copper concentrations with blood pressure was only observed in hypertensive participants taking thiazides, but not in those taking ACEI, CCB, ARB or BB. In addition, a 1SD increase in serum copper was non-significantly associated with 1.16 (0.97–1.37)-fold increased risk of hypertension in multivariable analysis (*p* = 0.096). As shown in Table S2, compared with the non-hypertension group, serum copper concentrations were significantly increased in patients using thiazides.

### 3.5. Subgroup and Sensitivity Analysis

**Figure 2:**
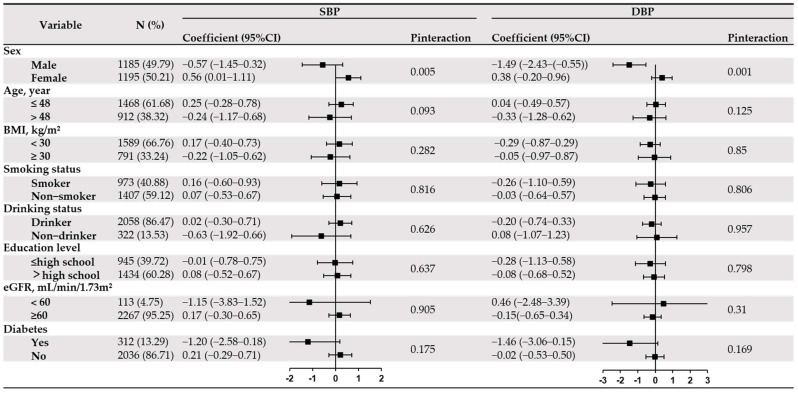
Stratified analyses by potential effect modifiers for the association between serum copper and blood pressure levels in non-hypertension. Notes: Black dots represent point estimates and error bars, 95% confidence interval. Models were adjusted for sex, age, education, smoking status, drinking status, BMI, eGFR, HDL-C, diabetes status, and CAD. The age was categorized into two subgroups (≤48 or >48 years) by the median value (48 year), BMI was categorized into two subgroups (<30 or ≥30 kg/m^2^), based on WHO criteria of weight, eGFR was cate-gorized into two groups (<60 or ≥60 mL/min/1.73 m^2^). Color should not be used for Figure 2 in print. In sensitivity analyses (Table S1), the relationship between serum copper and hypertension risk, as well as blood pressure levels, remained unchanged when excluding the subjects with eGFR < 60 mL/min/1.73 m^2^. Similar findings were also seen among participants in the range of 1–99% of serum copper concentration.

To explore the interactive effects on the association, we conducted subgroup analysis through a series of strata variables in Figure 2. Significant interactions with sex (all P interaction < 0.01) for SBP, as well as DBP, were found. There was no interaction between serum copper and other stratified variables except gender (all P interaction > 0.05).

## 4. Discussion

To the best of our knowledge, this is the first study to highlight the possible effects of antihypertensive drug use on the correlation between the serum copper and blood pressure. The main findings of the present study are as follows: serum copper levels were not significantly related to high blood pressure on either a categorical or continuous scale. The apparent-looking association of serum copper with hypertension could be attributed to antihypertensive drugs use.

In the untreated hypertension group, we did not find serum copper–hypertension associations in the multivariable analysis on a continuous or categorical scale. Since these patients were less likely to be prescribed antihypertensive drugs, our results could be deemed more reliable without the confounding effects of these medications. A small, matched, case-control study enrolling only 58 individuals found no statistical correlation between serum copper concentration and the risk of untreated hypertension. Similarly, another study with a large sample size enrolled only male populations and also discovered no significant difference in serum copper between untreated and non-hypertensive people [15]. These studies supported the findings revealed in the current study that serum–hypertension associations did not exist.

In non-hypertension individuals, there was most likelihood to observe the association of serum copper with blood pressure levels (as a continuous variable) if present. However, we also did not reveal the serum copper–blood pressure association on a continuous or categorical scale either in the univariate or multivariable analysis. It has been reported that in univariate analysis, serum copper concentration was not correlated with systolic and diastolic blood pressure levels after the exclusion of antihypertensive drug users [16]. A recent study that focused on the relationship between serum copper and blood pressure levels in 443 children aged 6–9 years also showed that serum copper was not related with either systolic or diastolic blood pressure in multivariable-adjusted models [17]. These findings from small-sized sample studies support our key findings. Furthermore, a Mendelian randomization study showed that high concentrations of genetically predicted copper were not appreciably associated with diastolic blood pressure levels in univariate analysis, which strongly supported our findings from a genetic perspective.

For the treated hypertension, we first observed the relationship of serum copper with hypertension in univariate analysis or in multivariable analysis without including antihypertensive drug use as a covariate. Very interestingly, this association finally disappeared after further adjusting the covariate of antihypertensive drug use in the multivariable analysis. Therefore, serum copper–hypertension association was greatly affected by the antihypertensive medications. Prior studies reporting significant associations of serum copper with hypertension largely did not include antihypertensive medications as a covariate [4,5,18]. One study included antihypertensive medications as a covariate and also revealed no correlation between serum copper and hypertension upon multivariable analysis, supporting our findings, although different hypertension diagnosis criteria were applied [7]. Studies have reported that the use of ACEI, ARB, BB, and CCB alone could not significantly change serum copper levels, and our findings also suggest that these four drugs have a weak effect on the relationship between copper and hypertension [19,20,21]. In addition, taking thiazide was particularly cofounding this association. One study also found that eight patients had a significant increase in plasma copper levels after 16 weeks of thiazide diuretic treatment [22].

This study had several limitations. First, this study is cross-sectional in design, and the confounding was unable to be avoided. Thus, this finding should be hypothesis-generating. Second, the antihypertensive drug-specific group is small in sample size, although this study includes 3376 subjects, a large sample size powered enough to indicate the absent associations of serum copper with blood pressures. Thus, more participants should be enrolled to study the serum copper–hypertension association in the antihypertensive drug-specific group so as to replicate our findings in the current study. Third, a reverse causality could bias our results, which are, thus, needed to be confirmed in randomized trials.

## 5. Conclusions

In the present study, we discovered that serum copper concentration was not related with hypertension or blood pressure. Antihypertensive drug use had a great impact on the serum copper levels and distorted its association with blood pressure levels. Identification of a novel risk factor for hypertension should be cautious in the absence of information on antihypertensive drug use.

## Figures and Tables

**Table 1:** Characteristics of the study population (NHANES, 2011–2014).

		Quartiles of Serum Copper, μmol/L					
Variables	Total	Q1 (<15.59)	Q2 (15.59–17.96)	Q3 (17.96–21.02)	Q4 (>21.02)	*P*	*P* for Trend
	*n* = 3376	*n* = 846	*n* = 850	*n* = 838	*n* = 842		
Age, year	48.00 (34.00–63.00)	42.00 (30.00–59.00)	49.00 (33.00–64.00)	53.00 (39.00–64.00)	50.00 (34.00–63.00)	<0.001	<0.001
Sex, % (n)						<0.001	<0.001
Male	49.61% (1675)	77.66% (657)	61.29% (521)	41.77% (350)	17.46% (147)		
Female	50.39% (1701)	22.34% (189)	38.71% (329)	58.23% (488)	82.54% (695)		
Education level, % (n)						<0.001	<0.001
Below high school	22.38% (755)	17.73% (150)	22.03% (187)	25.54% (214)	24.26% (204)		
High school	21.19% (715)	19.39% (164)	23.09% (196)	21.12% (177)	21.17% (178)		
Above high school	56.43% (1904)	62.88% (532)	54.89% (466)	53.34% (447)	54.58% (459)		
Smoking, % (n)						<0.001	0.075
Never	56.92% (1921)	61.23% (518)	54.24% (461)	51.49% (431)	60.69% (511)		
Former	23.29% (786)	22.58% (191)	26.12% (222)	24.85% (208)	19.60% (165)		
Current	19.79% (668)	16.19% (137)	19.65% (167)	23.66% (198)	19.71% (166)		
Drinking, % (n)						0.034	0.001
Never	15.24% (466)	13.73% (106)	13.05% (100)	15.80% (121)	18.44% (139)		
Former	16.38% (501)	14.64% (113)	17.49% (134)	16.84% (129)	16.58% (125)		
Current	68.38% (2091)	71.63% (553)	69.45% (532)	67.36% (516)	64.99% (490)		
BMI, kg/m^2^	28.97 ± 7.08	26.52 ± 5.07	28.16 ± 6.01	29.70 ± 7.19	31.50 ± 8.59	<0.001	<0.001
eGFR, mL/min/1.73 m^2^	94.14 ± 23.45	95.26 ± 22.86	94.19 ± 23.28	91.91 ± 23.02	95.18 ± 24.50	0.011	0.459
HDL-C, mmol/L	1.36 ± 0.40	1.30 ± 0.36	1.35 ± 0.41	1.38 ± 0.41	1.43 ± 0.41	<0.001	<0.001
Diabetes, % (n)	18.95% (628)	15.49% (129)	16.91% (141)	20.76% (170)	22.71% (188)	<0.001	<0.001
CAD, % (n)	6.48% (218)	5.92% (50)	7.21% (61)	7.17% (60)	5.62% (47)	0.41	0.801
SBP, mmHg	123.11 ± 17.94	121.43 ± 16.28	123.03 ± 18.04	124.58 ± 18.74	123.45 ± 18.51	0.005	0.092
DBP, mmHg	70.17 ± 12.74	70.95 ± 11.26	70.36 ± 12.75	69.41 ± 13.64	69.93 ± 13.20	0.097	0.275
Hypertension, % (n)	29.50% (996)	21.63% (183)	28.35% (241)	33.89% (284)	34.20% (288)	<0.001	<0.001
Treated Hypertension, % (n)	20.43% (611)	12.76% (97)	19.55% (148)	23.59% (171)	26.03% (195)	<0.001	<0.001
Untreated Hypertension, % (n)	13.92% (385)	11.48% (86)	13.25% (93)	16.94% (113)	14.37% (93)	0.027	0.030

Notes: BMI = body mass index; eGFR = estimated glomerular filtration rate; HDL-C = high-density lipoprotein cholesterol; CAD = coronary artery diseases, including coronary atherosclerotic heart disease, angina pectoris, and myocardial infarction; SBP = systolic blood pressure; DBP = diastolic blood pressure. Data are presented as mean ± standard deviation, median (interquartile range), or percentage (number). The Ps were calculated using the Kruskal–Wallis test, chi-square test or ANOVA test for different variables. The Ps for trend were performed using the STATA procedures Opartchi.

**Table 2:** Adjusted ORs (95% CI) of untreated hypertension by quartiles of serum copper level.

Serum Copper (μmol/L)	Cases	Non-Cases	Crude Model	Adjusted Model
			OR (95% CI)	
Q1 (<15.59)	86	663	1.00 (Ref)	
Q2 (15.59–17.96)	93	609	1.18 (0.86–1.61)	1.02 (0.74–1.42)
Q3 (17.96–21.02)	113	554	1.57 (1.16–2.13)	1.23 (0.88–1.72)
Q4 (>21.02)	93	554	1.29 (0.95–1.77)	1.08 (0.74–1.58)
*P*-trend			0.030	0.471
1SD increase	385	2380	1.07 (0.96–1.18)	1.04 (0.91–1.18)

Notes: The effect of serum copper on hypertension was expressed as an odds ratio (OR) and its 95% confidence interval (95% CI). The crude model was unadjusted. The adjusted model was further adjusted for age, sex, education, smoking status, drinking status, BMI, eGFR, HDL-C, diabetes status, and CAD. Ref = reference group.

**Table 3:** Association of serum copper concentration with systolic or diastolic blood pressure for non-hypertension.

Serum Copper (μmol/L)	Crude Model	Adjusted Model
	β (95% CI)	
SBP		
Q1 (<15.59)	1.00 (Ref)	
Q2 (15.59–17.96)	−0.54 (−1.78–0.70)	−0.92 (−2.07–0.23)
Q3 (17.96–21.02)	0.38 (−0.89–1.65)	−0.05 (−1.30–1.20)
Q4 (>21.02)	−1.29 (−2.56–(−0.02))	−0.48 (−1.83–0.88)
*P*-trend	0.161	0.775
1SD increase	−0.43 (−0.87–0.02)	0.06 (−0.40–0.53)
DBP		
Q1 (<15.59)	1.00 (Ref)	
Q2 (15.59–17.96)	−1.44 (−2.63–(−0.25))	−1.42 (−2.62–(−0.22))
Q3 (17.96–21.02)	−0.91 (−2.13–0.31)	−0.95 (−2.26–0.35)
Q4 (>21.02)	−1.23 (−2.45–(−0.01))	−1.28 (−2.70–0.13)
*P*-trend	0.092	0.129
1SD increase	−0.20 (−0.63–0.23)	−0.17 (−0.66–0.32)

Notes: The effect of vanadium on systolic and diastolic blood pressure was expressed as a regression coefficient and its 95% CI. The crude model was unadjusted. The adjusted model was further adjusted for age, sex, education, smoking status, drinking status, BMI, eGFR, HDL-C, diabetes status, and CAD. Ref = reference group.

**Table 4:** Associations of serum copper concentrations with the prevalence across different subgroups.

Outcomes			Categorical Models					Continuous Models	
	Cases	Non-Cases	Q1	Q2	Q3	Q4	*P* for Trend	1SD Increase	*p*-Value
Treated Hypertension	611	2380	1.00 (Ref)	1.36 (0.99–1.89)	1.49 (1.07–2.07)	1.83 (1.29–2.60)	0.001	1.22 (1.09–1.38)	0.001
Treated Hypertension ^a^	611	2380	1.00 (Ref)	1.36 (0.88–2.10)	1.35 (0.87–2.09)	1.56 (0.98–2.47)	0.088	1.16 (0.97–1.37)	0.096
Antihypertensive use									
ACE-I	85	2380	1.00 (Ref)	0.85 (0.42–1.74)	1.54 (0.79–2.99)	1.55 (0.75–3.20)	0.109	1.21 (0.93–1.57)	0.151
ARB	38	2380	1.00 (Ref)	1.26 (0.49–3.26)	0.88 (0.32–2.42)	0.47 (0.14–1.52)	0.157	0.71 (0.45–1.11)	0.136
BB	43	2380	1.00 (Ref)	5.36 (1.52–18.86)	3.05 (0.78–11.89)	5.00 (1.30–19.28)	0.094	1.41 (0.99–2.00)	0.053
CCB	34	2380	1.00 (Ref)	1.54 (0.88–2.68)	1.84 (1.05–3.21)	1.71 (0.94–3.13)	0.082	1.12 (0.91–1.37)	0.275
Thiazides	37	2380	1.00 (Ref)	5.54 (0.67–46.06)	5.59 (0.67–46.89)	15.74 (1.92–129.02)	0.001	1.72 (1.26–2.36)	0.001

Notes: ACE-I = angiotensin-converting enzyme inhibitor; ARB = angiotensin receptor blocker; BB = beta-blocker; CCB = calcium channel blocker; thiazides = thiazide and thiazide-like diuretics. ^a^ Model was further adjusted for antihypertensive drugs. Participants treated with other types of antihypertensive drugs were not included. Ref = reference group.

## Data Availability

This study used publicly available datasets from the NHANES database. The data can be found at https://www.cdc.gov/nchs/nhanes/index.htm (accessed on 5 July 2023).

## Supplementary Materials

The following supporting information can be downloaded at: https://www.mdpi.com/article/10.3390/jcdd10100432/s1, Figure S1: Serum copper distributions; Table S1: Sensitivity analysis for the associations of serum copper concentrations with hypertension.; Table S2: Hypertensive patients’ serum copper concentrations across different subgroups.
