# Supplementary material for: Serum Copper Concentrations, Effect Modifiers and Blood Pressure: Insights from NHANES 2011–2014
Source: J Cardiovasc Dev Dis. 2023 Oct 18;10(10):432. doi: 10.3390/jcdd10100432 (PMC10607875; doi:10.3390/jcdd10100432)
Supplement: Supplementary file 1 [file jcdd-10-00432-s001.zip › jcdd-2530970-supplementary.pdf]

Supplementary Contents

Figure S1. Serum copper distributions.

Table S1. Sensitivity analysis for the associations of serum copper concentrations with hypertension.

Table S2. Hypertensive patients' serum copper concentrations in different subgroups.

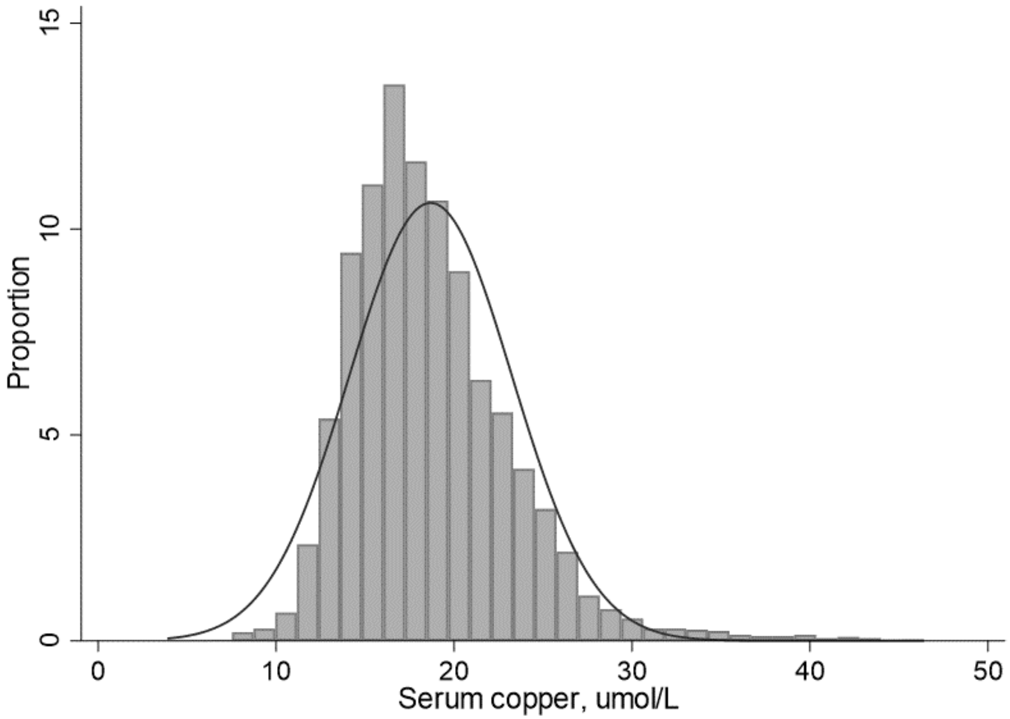


**Figure S1.** Serum copper distributions

**Table S1.** Sensitivity analysis for the associations of serum copper concentrations with hypertension.

| | Categorical models | | | | | Continuous models | |
|---|---|---|---|---|---|---|---|
| **Outcomes** | **Q1** | **Q2** | **Q3** | **Q4** | ***P* for trend** | **1SD increase** | ***P*–value** |
| Excluding participants with eGFR<60 mL/min/1.73m² | | | | | | | |
| Untreated Hypertension | 1.00 (ref) | 1.05 (0.75–1.47) | 1.17 (0.83–1.66) | 1.10 (0.75–1.63) | 0.515 | 1.04 (0.91–1.20) | 0.557 |
| Treated Hypertension | 1.00 (ref) | 1.31 (0.92–1.89) | 1.42 (0.99–2.05) | 1.76 (1.19–2.60) | 0.006 | 1.20 (1.05–1.38) | 0.008 |
| Treated Hypertension [a] | 1.00 (ref) | 1.42 (0.86–2.32) | 1.33 (0.81–2.17) | 1.69 (0.99–2.88) | 0.092 | 1.16 (0.95–1.42) | 0.154 |
| SBP | 1.00 (ref) | −0.67 (−1.84–0.50) | 0.26 (−1.01–1.53) | −0.19 (−1.57–1.20) | 0.879 | 0.18 (−0.30–0.65) | 0.466 |
| DBP | 1.00 (ref) | −1.81 (−3.03–(−0.59)) | −1.23 (−2.55–0.09) | −1.48 (−2.92–(−0.03)) | 0.088 | −0.15 (−0.65–0.34) | 0.542 |
| Excluding outliers (beyond the range of 1%–99%) of the serum copper | | | | | | | |
| Untreated Hypertension | 1.00 (ref) | 1.01 (0.72–1.41) | 1.21 (0.87–1.70) | 1.07 (0.73–1.56) | 0.515 | 1.03 (0.88–1.20) | 0.695 |
| Treated Hypertension | 1.00 (ref) | 1.35 (0.97–1.88) | 1.48 (1.06–2.06) | 1.82 (1.28–2.60) | 0.001 | 1.25 (1.07–1.46) | 0.004 |
| Treated Hypertension [a] | 1.00 (ref) | 1.41 (0.91–2.19) | 1.41 (0.90–2.19) | 1.59 (1.00–2.53) | 0.083 | 1.16 (0.93–1.43) | 0.186 |
| SBP | 1.00 (ref) | −0.97 (−2.13–0.19) | −0.13 (−1.39–1.13) | −0.77 (−2.15–0.61) | 0.506 | −0.15 (−0.70–0.41) | 0.604 |
| DBP | 1.00 (ref) | −1.51 (−2.73–(0.30)) | −1.05 (−2.37–0.27) | −1.52 (−2.96–(−0.07)) | 0.077 | −0.44 (−1.03–0.14) | 0.134 |

Notes: SBP=systolic blood pressure; DBP=diastolic blood pressure. [a] Model was further adjusted for antihypertensive drugs. Participants treated with other types of antihypertensive drugs were not included. Ref=reference group.

**Table S2.** Hypertensive patients' serum copper concentrations in different subgroups.

| **Outcome** | **serum copper concentration, μmol/L** | ***P*–value** |
|---|---|---|
| Non–hypertension (N=2380) | 18.45 ± 4.61 | Ref |
| Untreated Hypertension (N=385) | 18.67 ± 3.93 | 0.376 |
| Treated Hypertension (N=611) | 19.53 ± 4.34 | <0.001 |
| Antihypertensive use | | |
| ACE–I (N=85) | 19.17 ± 3.58 | 0.154 |
| ARB (N=38) | 18.24 ± 3.97 | 0.780 |
| BB (N=43) | 19.73 ± 4.51 | 0.071 |
| CCB (N=34) | 18.60 ± 3.44 | 0.850 |
| Thiazides (N=37) | 21.72 ± 4.83 | <0.001 |

Notes: ACE–I=angiotensin–converting enzyme inhibitor; ARB=angiotensin receptor blocker; BB=Beta–blocker; CCB=calcium channel blocker; Thiazides=thiazide and thiazide–like diuretics. *P*–values were calculated by Student's t–test, all group was set Non–hypertension as reference group. Ref=reference group.
